# Adipose-derived stem cells for treatment of chronic ulcers: current status

**DOI:** 10.1186/s13287-018-0887-0

**Published:** 2018-05-15

**Authors:** Jens Selch Holm, Navid Mohamadpour Toyserkani, Jens Ahm Sorensen

**Affiliations:** 1 Department of Plastic Surgery, Odense University Hospital, Odense, Denmark; 2 Department of Plastic Surgery, Roskilde Hospital, Roskilde, Denmark

**Keywords:** Chronic ulcers, Chronic wounds, Adipose-derived stem cells, Stromal vascular fraction

## Abstract

Chronic ulcers remain a difficult challenge in healthcare systems. While treatment options are limited, stem cells may be a novel alternative. Adipose-derived stem cells (ADSC) have become increasingly popular compared with bone marrow-derived stem cells as they are far easier to harvest. To summarize the current status of treating chronic ulcers with ADSC, this systematic review includes all clinical trials on the subject from PubMed and EmBase, as well as all registered clinical trials on ClinicalTrials.Gov. A total of nine clinical trials and fourteen registered trials were included. The studies were significantly different in terms of study design and patient population, and the overall quality of the studies was low to moderate. Despite the overall low study quality and the significant differences between the studies, some conclusions were consistent: ADSCs are safe, improve the healing of chronic ulcers, and reduce pain. As these results are consistent despite the shortcomings of the studies, they appear to highlight the efficacy of ADSCs in the treatment of chronic ulcers. Larger numbers of higher quality studies are needed to determine the precise role of ADSCs in treating chronic leg ulcers.

## Background

Chronic leg ulcers (CLU) are a common and complicated disease to treat [1], and result in high morbidity [2] and significantly reduced quality of life [3]. Normal wound healing consists of four overlapping phases: hemostasis, inflammation, proliferation, and remodeling [4]. Most ulcers heal when the cause is eliminated and the ulcer is treated with standard wound care. Some ulcers, however, are for various reasons locked in the inflammatory stage and do not heal [5]. Regarding CLUs, most of the ulcers are caused by venous insufficiency or arterial ischemia, often secondary to diabetes, but some CLUs do not have an apparent underlying condition [6].

CLUs are a challenge for the physician, a significant physical and psychological setback for the patient, and a heavy burden on the healthcare systems. Thus, an American study reported an average cost of treating chronic venous leg ulcers of $9685 per patient per year [7]. This reflects that these patients are difficult to treat, as the available treatment options are limited, leaving patients with a chronic condition severely affecting their quality of life. CLUs result in substantial use of the resources of healthcare systems regarding materials, hospital appointments, reduced working capability for the patients, and impairment of the patient in general [8].

Treatment with stem cells might be a new treatment option for these patients. Stem cells [9] have the potential to differentiate into numerous types of cells [10]. Over the last decade, stem cell therapy has shown great potential in the treatment of a variety of different conditions, such as orthopedic disorders, inflammatory diseases, hepatic failure, and autoimmune disorders [11, 12]. At the time of writing, adverse events have not been reported; therefore, stem cell treatment is currently regarded as safe [13]. A review [14] including more than 1400 patients found a favorable safety profile of adipose-derived stem cells (ADSC), but also highlighted the poor quality of most studies in regard to registering adverse events.

Adipose tissue is an excellent source of autologous ADSC and can be harvested easily compared with bone marrow-derived stem cells (BMSC) [15]. Adipose tissue has in recent years surpassed bone marrow as the preferred source of mesenchymal stem cells [16]. Besides being abundant, far easier to harvest, and with a lower risk of complications for the patient, adipose tissue additionally contains about 40 times more stem cells than bone marrow [17]. A simple liposuction of the abdomen or inner thigh performed under local or general anesthesia is sufficient to harvest the required number of ADSC without any significant risk of complications [18]. ADSC can be either freshly isolated or cultured. The culturing takes several days or weeks, and cannot be performed as a same-day procedure.

Stem cells are a heterogeneous pool of cells with numerous capabilities [19]. They possess anti-inflammatory and neoangiogenic effects, secrete numerous growth factors, and can differentiate into various cells types [20]. Many of these are known to be involved in the complex healing of wounds [21], although the exact capabilities and mechanisms of action of stem cells in wound healing are not yet fully understood. Research suggests that stem cells work though two mechanisms of action: firstly they attenuate the general inflammatory response and, secondly, they transform into cells involved in wound healing such as fibroblasts, myofibroblasts, antigen presenting cells, endothelial progenitor cells, and so forth [22].

Freshly isolated ADSC are far more heterogeneous compared with the quite homogeneous cells harvested from cultured ADSC [23]. The greater variety of cell types in the freshly isolated ADSC could have a significant advantage in wound healing, compared with the far more limited number of different cells in cultured ADSC. In ADSC, endothelial, hematopoietic, and pericytic lineages represent 10–20%, 25–45%, and 3–5%, respectively, of the total nucleated cells.

Several methods of isolating ADSC have been reported [16, 24]. The most common is enzymatic isolation. Some techniques use simple centrifugation and vibration to isolate a stem cell pellet. Others are more complex and involve, for example, enzymatic (collagenase) dissolving of the adipose tissue. Fully automatic systems also exist. The different methods have advantages and disadvantages regarding the time required for the procedure, the need for advanced equipment/specially trained personnel, and financial cost. Data describing the efficacy of various methods are not available; therefore, no standardized method exists [25]. In addition, the optimal method of application of ADSC is still undecided.

Various animal studies have documented the positive effect of ADSC in accelerating healing of chronic ulcers [19]. The clinical translation is ongoing, with several clinical studies already published. The aim of this review is to describe the available data on the treatment of CLU with autologous ADSC by identifying published human studies and ongoing/registered clinical trials on the matter.

## Data acquisition

**Fig. 1:**
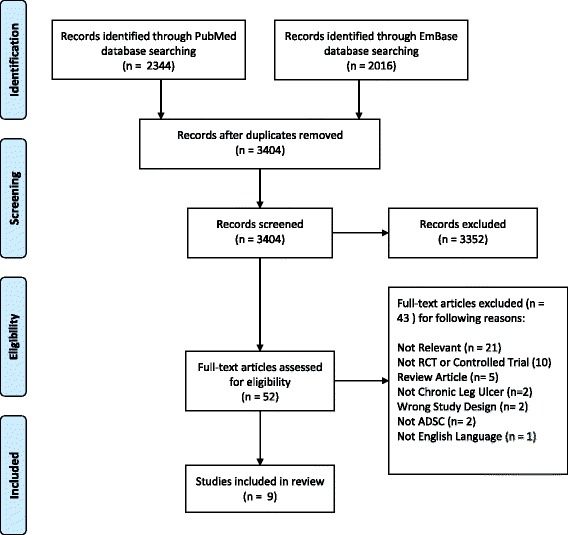
Search flow diagram. ADSC, adipose-derived stem cells; RCT, randomized controlled trial

To identify the relevant clinical trials, a search was performed on PubMed and EmBase for all human studies in English on ADSC in the treatment of chronic ulcers (Fig. 1) according to the PRISMA statement [26]. The search was performed in December 2017 independently by both the first and second author of this article by using the search terms: (“adipose-derived stem cells” OR “adipose stem cells” OR “stromal vascular fraction” OR “mesenchymal cells” OR “stromal cells”) AND (“wound healing” OR “ulcer”). Articles from 1 January 1995 to December 2017 were included. A similar search was performed on EmBase.

In total, 3404 articles were identified after duplicates were removed (*n* = 956) using Covidence [27]. The titles and abstracts were screened, and 3352 articles not relevant to the subject were excluded, leaving 52 articles that were potentially relevant and the full articles were obtained for further review. Of these, 43 studies were excluded (see Fig. 1 for more detail) and a total of nine clinical studies were identified and included in the review (Table 1). Their reference lists were evaluated manually for additional studies. Author/year, title, cause of ulcer, patient population, study design, type of ADSC, application method, primary endpoints, follow-up duration, and conclusions were recorded from the included studies. For a full overview of the results, see Table 1.

**Table 1:** Published Clinical Trials

Author, year, and country	Title	Cause of ulcers	Patients	Randomization and blinding	Type of ADSC	Application method	Primary endpoint	Follow-up	Conclusion
Han et al. [28], 2010, Korea	The treatment of diabetic foot ulcers with uncultured, processed lipoaspirate cells: a pilot study	Peripheral artery disease (diabetes 100%)	28 cases 26 controls Total 54	Yes, open label	Adipose-derived stem cell (ADSC) pellet isolated using collagenase and centrifugation. Donor site: abdomen 4.0 × 10^6^ to 8.0 × 10^6^ cells	Same-day procedure. Dispersed onto the wound and sealed with fibrinogen, thrombin, and Tegaderm	Wound closure rate	2 months	100% of wounds healed in 8 weeks in the case group, 62% in the control group. No adverse events
Lee et al. [29], 2012, Korea	Safety and effect of adipose tissue-derived stem cell implantation in patients with critical limb ischemia: a pilot study	Peripheral artery disease. Thromboangiitis obliterans (80%) and diabetes (20%). 80% with chronic wounds	15 cases 0 controls Total 15	No, open label	Digested using collagenase and centrifugation. Cultured. Donor site: abdomen 3.0 × 10^8^ cells.	60 intramuscular injections under spinal anesthesia. Time from harvest to injection not mentioned in study	Absence of adverse events. Formation of collateral networks. Secondary endpoint: pain, amputation, healing of wounds	6 months	Chronic wounds healed in 66.7% of patients. At 6 months, improvement in pain rating and in claudication walking distance. Five patients required minor amputation during follow-up, and all amputation sites healed completely. No adverse events
Marino et al. [30], 2013 Italy	Therapy with autologous adipose-derived regenerative cells for the care of chronic ulcer of lower limbs in patients with peripheral arterial disease	Peripheral artery disease	10 cases 10 controls Total 20	No, open label	Freshly isolated using Celution® 800/CRS. Donor site: abdomen/inner thigh. 260 g lipoaspirate 1.5 × 10^6^ cells	Same-day procedure. 5 mL injected in 1 cm deep injections around the ulcer under peripheral block of the sciatic nerve using a 10-mL syringe and 21-gauge needle	Complete healing of the ulcer	3 months	Reduction in size, depth, and pain of all cases compared with controls. 6 of 10 cases had total healing, none in control group. No adverse events
Bura et al. [31], 2014 France	Phase I trial: the use of autologous cultured adipose-derived stroma/stem cells to treat patients with non-revascularizable critical limb ischemia	Peripheral artery disease	7 cases 0 controls Total 7	No, open label	Isolated using collagenase, centrifugation and then cultured. Donor site: abdomen. (30 g) 60 mL lipoaspirate 10^8^ cells	14 days after liposuction. 26 mL injected in 30 intramuscular injections (15 in each muscle) into the internal and external gastrocnemius and anterior compartment of the ischemic leg using a 23-gauge needle	Improvement of wound healing	6 months	Ulcer size, ulcer number, and pain reduced. Improved transcutaneous saturation. No adverse events
Raposio et al. [32], 2016 Italy	Adipose-derived stem cells added to platelet-rich plasma for chronic skin ulcer therapy	Venous (45%), ischemic (42%), diabetic (10%) and post-traumatic (3%)	16 cases 24 controls Total 40	Yes, open label	e-PRP from 42 cm^3^ of peripheral blood combined with ADSC from 80 mL of abdominal fat vibrated at 600 vibrations/min for 6 min and centrifuged at 52 g for 6 min. 5 × 10^5^ cells	Same-day procedure. 5 mL injected in multiple injections around and under the ulcer using a 10-mL syringe	Wound closure rate	18 months	Similar healing rates. Wound closure rates higher in case group. No adverse events
Carstens et al. [33], 2017, Nicaragua	Non-reconstructable peripheral vascular disease of the lower extremity in ten patients treated with adipose-derived stromal vascular fraction cells	Peripheral artery disease (3 diabetes, 4 atherosclerosis, and 3 both)	10 cases 0 controls Total 10	No, open label	Fresh, non-fractioned, non-cultured. Enzymatic congestion using collagenase and centrifugation. Donor site: abdomen 250–350 cm^3^ fat. 19.1 to 157.8 × 10^6^ cells	3–4 mL administered using a 26-gauge needle into the plane between the gastrocnemius and soleus muscles in a pattern of injections (22 per muscle, 11 in the external and 11 in the internal gastrocnemius, each one 1.5 cm to 2 cm apart) of equal volume each (0.5 ml), on either side of the midline	Wound closure rate, pain	18 months	4 of 6 wounds closed within 9 months, one patient had a healing wound when she died at 4 months and 1 patient had a skin graft to close the wound at 5 months. Reduced pain in all patients. No adverse events
Chopinaud et al. [34], 2017 France	Autologous adipose tissue graft to treat hypertensive leg ulcer: a pilot study	Hypertensive	10 cases 0 controls Total 10	No, open label	LipoStructure®. Freshly purified fat using centrifugation at 3000 rpm for 3 min.	Same-day procedure. Multiple injections around and under the ulcer with 0.8-mm cannula	Wound closure rate	6 months	73.2% median closure rate at 3 months, 93.1% at 6 months. Reduced fibrin, necrosis and pain. Increased granulation. No adverse events
Konstantinow et al. [35], 2017 Germany	Therapy of ulcus cruris of venous and mixed venous arterial origin with autologous, adult, native progenitor cells from subcutaneous adipose tissue: a prospective clinical pilot study	Arterial-venous (9 patients), venous (7 patients). 6 patients with diabetes	16 cases 0 controls Total 16	No, open label	The Transpose RT™ Processing Unit (TPU) (InGeneron Inc., Houston, TX, USA) 30 mL lipoaspirate. Donor site: abdomen. 9–15 × 10^6^ cells	Same-day procedure. 4 ml injected 5 to 10 mm deep into the central and bordering ulcer area using a 1-mL Luer-Lock syringe and a 24-gauge needle. Additionally, 2.5 mL applied on a collagen sponge onto the wound	Wound closure rate, pain	6 months	All venous patients and four of nine arterial-venous patients had 100% wound closure within 9–26 weeks. Reduced wound pain in all patients within days of treatment. No adverse events
Darinskas et al. [36], 2017 Lithuania	Stromal vascular fraction cells for the treatment of critical limb ischemia: a pilot study	Peripheral artery disease (7 patients with ulcers) (9 patients with diabetes)	15 cases 0 controls Total 15	No, open label	Uncultured ADSC isolated without collagenase using mechanical isolation (the fat minced using a metal mill and subsequent centrifugation) 40 mL lipoaspirate Donor site: abdomen	One or two 20-mL syringes with minimum of 20 million viable cells per syringe and a minimum of 30 injections per syringe. Intramuscular injections along the arteries. Secondary injections were performed 2 months after first application of cells	Wound closure rate, pain	12 months	All ulcers healed. Two patients had amputations. Reduced pain in all patients. 86.7% with improvement in walking distance. No adverse events

To identify past, ongoing, or future registered studies on ADSC in the treatment of chronic ulcers, a thorough search of ClinicalTrials.gov was performed. Using the search terms “stem cells” or “adipose” or “stromal cells” in combination with “ulcer” or “wound”, a total of fourteen studies were identified (Table 2).

**Table 2:** Registered Cinical Trials

Study	Type of stem cell and application method	Design	Condition	Trial institution	NCT number and duration period	Status
A) Safety and effect of adipose tissue derived mesenchymal stem cells implantation in patients with critical limb ischemia	Autologous adipose-derived stem cells (ADSC) from lipoaspirate (not further detailed). Intramuscular injection.	Allocation: non-randomized Control group: none Blinding: none (open label) Follow-up: 3 months Estimated enrollment: 20	Critical limb ischemia	Pusan National University Hospital, Korea	NCT01663376 January 2009 to April 2011	Status: completed. Study published in *Circulation Journal* Vol. 76, July 2012 [29]
B) The role of lipoaspirate injection in the treatment of diabetic lower extremity wounds and venous stasis ulcers	Autologous lipoaspirate with no further ADSC isolation. Implantation in single tunnels radially around each wound spaced at 5–10 mm apart and approximately 3–5 cm in length	Allocation: randomized Control group: sterile tumescence solution Blinding: single (outcomes assessor) Follow-up: 12 months Estimated enrollment: 250	Diabetic and venous stasis wounds	Washington DC Veterans Affairs Medical Center, Columbia, USA	NCT00815217 February 2009 to February 2010	Status: unknown. The recruitment status of this study is unknown. The completion date has passed and the status has not been verified in more than 2 years Last update: December 2008
C) Application of cell regeneration therapy with mesenchymal stem cells from adipose tissue in critical chronic ischemic syndrome of lower limbs (CLI) in nondiabetic patients.	Autologous ADSC (not further detailed). Infusion of mesenchymal stem cells from adipose tissue administered intraarterially	Allocation: randomized three armed (high vs. low-dose vs placebo) Control group: conventional treatment Blinding: none (open label) Follow-up: 12 months Estimated enrollment: 30 (10 in each arm)	Critical limb ischemia	Hospital San Lazaro and University Hospital Virgen Macarena, Sevilla, Spain	NCT01745744 February 2011 to December 2017	Status: this study is ongoing, but not recruiting participants. Last update: September 2017
D) Stem cell therapy for patients with vascular occlusive diseases such as diabetic foot	Autologous mesenchymal stem cells (not further detailed). Application method not detailed	Phase: 1 Allocation: non-randomized Blinding: none (open label) Control group: none Follow-up: 6 months Estimated enrollment: 20	Diabetic foot and lower limb ischemia	Chinese PLA General Hospital, China	NCT02304588 January 2013 to December 2015	Status: this study is currently recruiting participants. Last update: December 2014
E) Treatment of hypertensive leg ulcer by adipose tissue grafting (Angiolipo)	Autologous ADSC harvested from autologous lipoaspirate. (not further detailed). Application method not detailed	Phase: 1 Allocation: non-randomized Blinding: none (open label) Control group: none Follow-up: 6 months Estimated enrollment: 10	Hypertensive ulcers	University Hospital, Caen, France	NCT01932021 April 2013 to December 2014	Status: completed. Study published July 2017 in *Dermatology* [34]
F) Adipose derived regenerative cellular therapy of chronic wounds	Autologous ADSC from autologous lipoaspirate. (not further detailed). Multiple injections of ASC into the periphery and debrided surfaces of chronic wounds	Phase: 2 Allocation: non-randomized Blinding: none (open label) Control group: none Follow-up 3 months Estimated enrollment: 25	Chronic wounds	Tower Outpatient Clinic, Los Angeles, California, USA	NCT02092870 September 2013 to September 2015	Status: unknown. The recruitment status of this study is unknown. The completion date has passed and the status has not been verified in more than 2 years. Last update: March 2014
G) To evaluate the safety and efficacy of IM and IV administration of autologous ADMSCs for treatment of CLI	Autologous stromal vascular fraction and autologous adipose derived MSC (not further detailed). Injected intravenously and intramuscularly vs intramuscularly only	Allocation: randomized (autologous stromal vascular fraction vs autologous adipose derived MSCs) Blinding: none (open label) Primary purpose: treatment Control group: no treatment Follow-up 9 months Estimated enrollment: 60	Critical limb ischemia	Kasiak Research Pvt. Ltd., India	NCT02145897 August 2014 to August 2015	Status: unknown. The recruitment status of this study is unknown. The completion date has passed and the status has not been verified in more than 2 years. Last update: May 2014
H) A clinical study using adipose-derived stem cells for diabetic foot	Autologous ADSC from lipoaspirate (not further detailed). Injections to the wound	Allocation: randomized Control group: saline Blinding: none (open label) Follow-up: 3 months Estimated enrollment: 240	Peripheral vascular disease, ischemia, and diabetic foot	The Third Affiliated Hospital of Southern Medical University, Guangzhou, Guangdong, China	NCT02831075 January 2015 to December 2018	Status: this study is currently recruiting participants. Last update: April 2017
I) Adipose-derived stromal cells (ASCs) and pressure ulcers	Autologous ADSC from lipoaspirate (not further detailed). ADSC injected into a fibrin sealant and applied to the wound	Allocation: randomized Control group: placebo Blinding: quadruple (participant, care provider, investigator, outcomes assessor) Follow-up: 6 months Estimated enrollment: 12 (6 in each arm)	Stage 3 and 4 pressure ulcers	Mayo Clinic, Florida, USA	NCT02375802 July 2015 to July 2017	Status: this study is currently recruiting participants. Last update: September 2016
J) Effectiveness and safety of adipose-derived regenerative cells for the treatment of critical lower limb ischemia	Autologous ADSC extracted from lipoaspirate by enzymatic digestion (nor further detailed). 10 mL of autologous ADSC injected intramuscularly	Phase: 1 Allocation: non-randomized Blinding: none (open label) Control group: none Follow-up: 24 weeks Estimated enrollment: 9	Critical limb ischemia, arteriosclerosis obliterans, peripheral arterial disease Thromboangiitis obliterans, diabetic angiopathies	Central Clinical Hospital w/Outpatient Health Center of Business Administration for the President of Russian Federation, Russia	NCT02864654 July 2016 to July 2018	Status: this study is enrolling participants by invitation only. Last update: August 2016
K) Assessment of the efficacy and tolerance of sub-cutaneous re-injection of autologous adipose-derived REGEnerative Cells in the Local Treatment of Neuropathic Diabetic Foot ulcERs (REGENDER)	Autologous ADSC from lipoaspirate (not further detailed). Injections to the wound	Phase: 2 Allocation: non-randomized Blinding: none (open label) Control group: none Follow-up: 20 weeks Estimated enrollment: 45	Diabetic foot ulcer	Assistance Publique Hopitaux De Marseille, France	NCT02866565 February 2017 to November 2019	Status: not yet recruiting Last update: August 15, 2016
L) Healing chronic venous stasis wounds with autologous cell therapy	Autologous ADSC isolated from lipoaspirate by Transpose® RT System (InGeneron Inc., Texas, USA). Subcutaneous injection around the rim of the wound	Phase: 2 Allocation: randomized Blinding: none (open label) Control group: no treatment Follow-up: 12 months Estimated enrollment: 36 (24 cases, 12 controls)	Chronic venous stasis wounds	Sanford USD Medical Center, Sioux Falls, South Dakota, USA	NCT02961699 June 2017 to January 2020	Status: this study is currently recruiting participants Last update: August 2017
M) Clinical application of mesenchymal stem cells seeded in chitosan scaffold for diabetic foot ulcers	Autologous mesenchymal stem cell seeded in curcumin-loaded chitosan nanoparticles into collagen-alginate. Application method not detailed	Phase: 1 Allocation: non-randomized Blinding: none (open label) Control group: none Follow-up: 12 months Estimated enrollment: 40	Diabetic foot ulcer	Assiut University, Assiut, Republic of Egypt	NCT03259217 October 2017 to January 2019	Status: this study is not yet open for participant recruitment. Last update: August 2017
N) Safety of adipose-derived stem cell stromal vascular fraction	Autologous ADSC from lipoaspirate (not further detailed). Injections to the wound	Phase: 1 Allocation: non-randomized Blinding: none (open label) Control group: none Follow-up: 20 weeks Estimated enrollment: 10	Abnormally healing wounds, scars, soft tissue defects	Forest Hill Institute of Aesthetic Plastic Surgery, Toronto, Ontario, Canada	NCT02590042 October 2017 to January 2021	Status: this study is not yet open for participant recruitment. Last update July 2017

Study title, type of ADSC and application method, study design, cause of ulcer, trial institution, NCT number, duration period, and study status were recorded.

## Results

### Published clinical studies

The clinical trials included in the review were: study 1, Han et al. [28]; study 2, Kirana et al. [29]; study 3, Marino et al. [30]; study 4, Bura et al. [31]; study 5, Raposio et al. [32]; study 6, Carstens et al. [33]; study 7, Chopinaud et al. [34]; study 8, Konstantinow et al. [35]; and study 9, Darinskas et al. [36].

The data were collected as described in the Data acquisition section; for the full overview of the included studies and data, refer to Table 1.

#### Cause of chronic ulcers

All studies except study 3 included patients with peripheral artery disease (PAD). PAD was primarily a complication to diabetes (studies 1, 2, 6, 7, 8, and 9), thromboangiitis obliterans (study 5) or primary atherosclerosis (study 4). Studies 3 and 4 included patients with hypertensive and venous ulcers. Study 3 had only hypertensive/venous ulcers, whereas study 4 had 45% hypertensive/venous ulcers. Study 8 included 16 patients of which nine had arterial-venous disease and seven patients with venous disease.

#### Study design

The studies varied significantly in study design. No studies were blinded and only studies 1 and 5 were randomized. Three studies (1, 3, and 5) had control groups. The size of the groups ranged from 7 to 28 patients in the case groups and from 0 to 28 patients in the control groups, and the total number of patients included ranged from 7 to 54 patients. The follow-up period varied from 2 months (study 1) to 18 months (studies 5 and 6).

#### Type of ADSC

Different types of stem cells were used in the studies. Freshly isolated stem cells were used in studies 1, 3, 5, 6, 7, 8, and 9, and cultured cells were used in studies 2 and 4. All studies used adipose tissue harvested from the abdomen or inner thigh using liposuction.

Studies 1 and 6 isolated an ADSC pellet using collagenase and centrifugation. Studies 2 and 4 used cultured stem cells digested with collagenase and centrifuged. In study 3, a Celution 800® system isolated the ADSC. In study 5, e-PRP (platelet-rich plasma combined with ADSC isolated using vibration and centrifugation) was the type of ADSC investigated. Study 7 isolated the ADSC with the LipoStructure® [37] technique. Study 8 isolated the ADSC with the Transpose RT™ Processing Unit (TPU; InGeneron Inc., Houston, TX, USA).

Study 9 investigated uncultured ADSC isolated without collagenase using mechanical isolation (the fat was minced using a metal mill and subsequently centrifuged).

#### Application method

The application method varied from study to study. Topical application of the stem cells onto the wound was performed in study 1. Studies 2, 4, 6, and 9 injected the ADSC intramuscularly. In study 3, the stem cells were injected around the ulcer and, in studies 5 and 7, the stem cells were injected into and around the ulcer. Study 8 injected the ADSC into and around the ulcer, but also applied 2.5 mL of ADSC onto a sponge which was fixed on top of the ulcer.

#### Ulcer healing

All studies reported healing of the chronic ulcers to varying degrees. The results reported were:

- Study 1: 100% healing of the case group compared with 62% in the control group.
- Study 2: 66.7% of the chronic ulcers had healed at 6 months.
- Study 3: Six out of ten patients in the case group had total healing of the ulcer versus none in the control group.
- Study 4: A decrease in the number of ulcers and in ulcer size in all patients, except two patients where amputation was performed.
- Study 5: Reported similar total ulcer healing rates between the case and the control group (71% vs 68%), but a significantly higher wound closing rate in the case group (0.2287 cm^2^/day vs 0.0890 cm^2^/day, *p* = 0.0257).
- Study 6: Four of six wounds closed within 9 months. Regarding the remaining two patients, one patient had a wound in the granulation stage when she died of unrelated cardiac arrest at 4 months and the other patient had a successful skin graft to close the wound at 5 months, where the ulcer was in the granulation stage.
- Study 7: A 73.2% median closure rate at 3 months and 93.1% at 6 months.
- Study 8: All venous ulcers and four of nine arterial-venous ulcers healed. Complete wound closure was achieved within 9 to 26 weeks of ADSC treatment.
- Study 9: Seven patients with ulcers. All ulcers healed, although two patients required major amputation after which the amputation sites healed.

#### Study conclusions

The overall quality of the studies is low to moderate. The patient populations are limited in size and only a few studies are randomized. The lack of blinding in all studies as well as the limited randomization significantly increases the risk of bias. Quadruple blinding, larger study populations, matched control groups, and more homogenous studies would significantly improve the current research. Basic research investigating optimal dosage of stem cells and the administration route is also lacking.

Despite the shortcomings in terms of quality of the studies, and the studies being heterogeneous, some conclusions are consistent:

1. No studies report any adverse event of significance, if any at all.
2. In all studies examining wound-related pain, they all found a reduction in the sensation of pain following stem cell treatment (studies 2, 3, 4, 6, 7, 8, and 9).
3. All studies showed noteworthy progress in the healing of the chronic ulcers. In the studies with control groups, a significantly higher healing rate of the case groups compared with the control groups was seen.

### Registered clinical trials

All relevant clinical trials registered on www.ClinicalTrials.gov were included (in total fourteen studies; see Table 2 for full overview of the studies).

#### Status

A few studies were completed, and the results published (studies A and E), while most were either ongoing (C, H, I, J, and L), not yet recruiting patients (K, M, and N) or have exceeded the anticipated completion date considerably without an update for years (B, D, F, and G).

#### Study design

The study design varies from study to study. Studies B, C, G, H, I, and L are randomized, but only study I and B are blinded. Studies B, C, H, I, and L have control groups. Estimated enrollment of patients ranges from 9 (J) to 250 (B). The follow-up period ranges from 3 months (A, F, and H) to 12 months (B, C, L, and M).

#### Cause of chronic ulcers

Several causes of ulcers are included in the studies. Critical limp ischemia (A, C, G, and J), diabetic foot ulcers (D, K, and M), pressure ulcer (I), chronic venous stasis ulcers (L), and hypertensive ulcers (E) were the diagnoses involving a single underlying condition. Study B includes both diabetic and venous ulcers and studies D and H include diabetic foot ulcers and lower limp ischemia. Study J examines critical limp ischemia, thromboangiitis obliterans, and diabetic foot ulcers. Study F includes chronic wounds without further specification.

#### Type of ADSC

Most studies utilize autologous ADSC from lipoaspirate without further specification (studies A, C, D, E, F, G, H, I, K, M, and N). However, study B uses autologous lipoaspirate with no further ADSC isolation, study J uses ADSC extracted by enzymatic digestion, study L isolates the ADSCs using the Transpose® RT System, and study M involves stem cells seeded in curcumin-loaded chitosan nanoparticles into collagen-alginate.

#### Application method

Intramuscular injection is planned for studies A and J. Injection into or around the wound is planned in studies B, F, H, K, L, and N. Study C involves intra-arterial administration of the stem cells. In study I, the ADSC are injected into a fibrin sealant and applied to the wound. Studies D, E, and M do not explain how the stem cells are planned to be applied. Study G applies intravenous and intramuscular injection versus intramuscular injection only.

#### Summary

As in the published clinical studies in this field, the registered clinical studies differ significantly. No consistency was observed across isolation technique, application method, or dose of ADSC. Furthermore, several different causes of ulcer are being investigated and the study designs are different. A few of the studies have been published, some are ongoing, but some of the studies have surpassed their estimated completion date and the status is unknown. Whether these studies will ever be completed/published remains questionable. Not all ongoing clinical trials are necessarily registered on ClinicalTrials.gov, however, and there is no doubt that a considerable amount of research in this field is currently being conducted, and probably even more clinical trials than the registered trials are currently being conducted.

## Discussion

This systematic review concludes that current clinical studies report that ADSC are safe, improve the healing of chronic ulcers, and reduce pain. Interestingly, these findings are consistent despite the overall poor study quality, with a risk of significant bias and major diversity between the studies regarding study design, underlying conditions, methods of isolation, and methods of application. This finding might show that ADSC have a significant effect on chronic wounds of many etiologies and are not particularly dependent on isolation and administration techniques. On the other hand, one could argue that the effect seen is caused by the substantial risk of bias and not by the effect of the stem cells.

The published studies, as well as the ongoing studies, on the subject are all diverse in study design. This is because several key questions remain unanswered in the field of autologous ADSC in the treatment of chronic ulcers: What method of isolating ADSC is superior? What conditions can be treated? Which application method is best? What amount of stem cells is needed? To answer these questions, large blinded and randomized studies as well as additional basic research on the biology and capabilities of stem cells are needed. High-quality blinded, randomized studies in this field are still lacking, although a few appear to be ongoing. A significant amount of research in this field appears to be ongoing. Further studies, however, are needed to define the long-term safety and efficacy of ADSC.

In summary, BMSC and ADSC appear to have a positive effect on the healing of chronic wounds [38]. A systematic review and meta-analysis of BMSC in treatment of chronic leg ulcers [39] found that BMSC, like ADSC, are both safe and efficient. BMSC and ADSC seem to be alike in terms of differentiation capacities and immune-modulatory properties [40]. If this is true, ADSC would inarguably be the preferred type of stem cell due to the less invasive harvesting procedure needed to obtain them and the abundance of fat for harvesting compared with bone marrow. BMSC thus have no apparent advantages over ADSC. Automatic closed systems have now enabled ADSC treatment to be a fast and safe same-day procedure, making the treatment favorable but also costly. The high financial cost of standard chronic wound care means that, although stem cell treatment is expensive, it might be cheaper in the long-term and can potentially save society a substantial amount of resources if able to heal the chronic wound compared with life-long wound care. Some low-quality studies [41] report an effect of simply transplanting fat without isolation of the stem cells, which could be an alternative treatment option if resources are limited.

A limit to this study is the diversity of the studies included. The number of clinical trials in this field is still limited. Definitive conclusions on the matter are not possible, as large studies with high quality and low risk of bias are needed.

## Conclusion

In conclusion, ADSC appear to be safe and have a positive effect on the healing of chronic ulcers. Treatment options for chronic ulcers are currently extremely limited and few new treatments are under development. Treatment with ADSC, however, is a novel and very exciting new treatment for chronic ulcers and might soon earn a pivotal role in this treatment; future studies will define exactly what that role will be.
